# Supplementary material for: Tead1a Initiates Transcriptional Priming Through the TEAD1a/YAP‐Notch1‐Spi1/Cebpα Axis to Promote Neutrophil Fate
Source: Adv Sci (Weinh). 2025 Aug 28;12(41):e05441. doi: 10.1002/advs.202505441 (PMC12591127; doi:10.1002/advs.202505441)
Supplement: Supplementary file 4 — Supplementary Dataset [file ADVS-12-e05441-s004.zip › 3-4.zip!/3-4/02-4.cluster/cluster11/cluster11_featureplot.pdf]

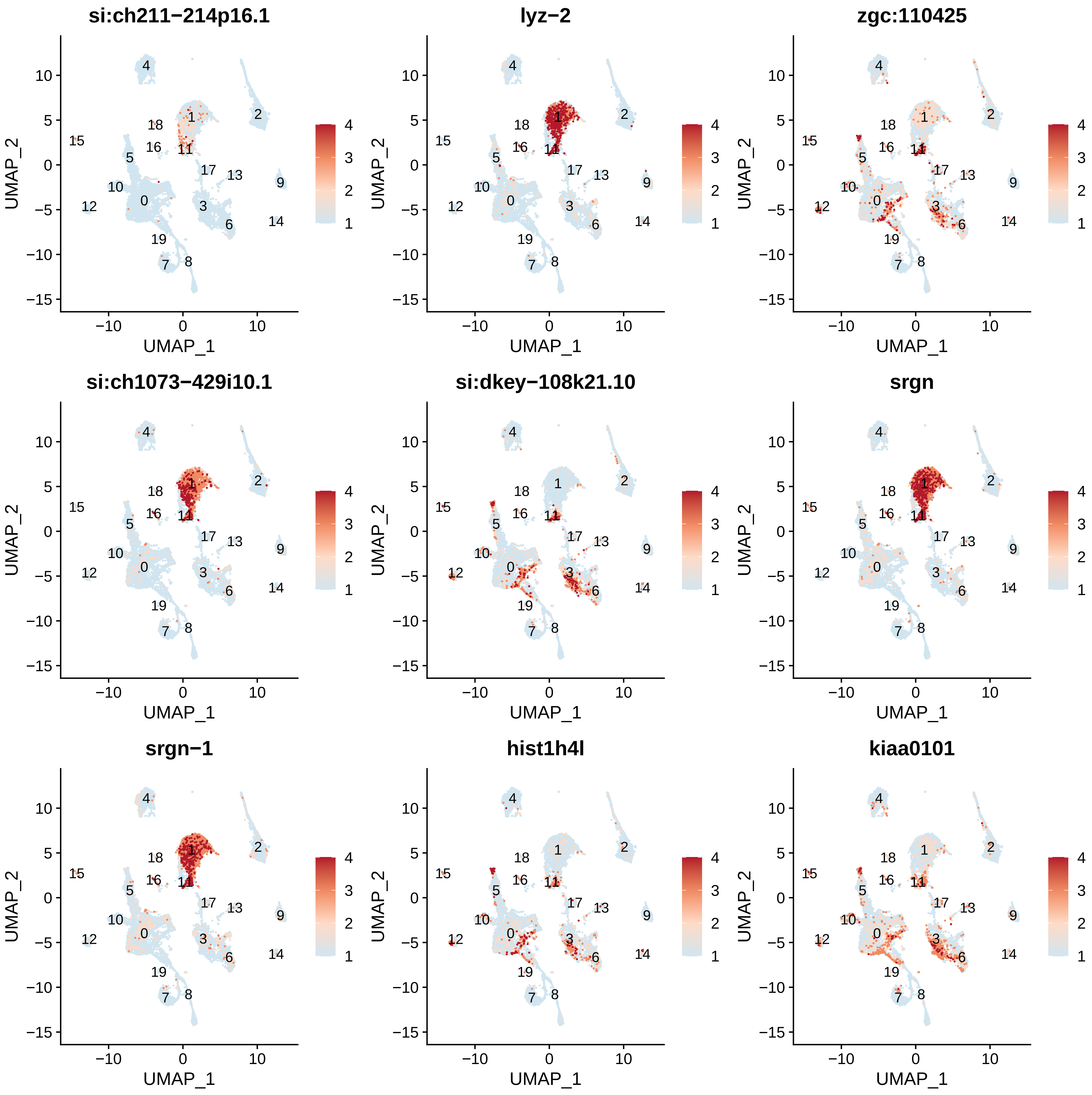
si:ch211−214p16.1
lyz−2
zgc:110425
si:ch1073−429i10.1
si:dkey−108k21.10
srgn
srgn−1
hist1h4l
kiaa0101
UMAP_1
UMAP_2
